# Embodied cognition, abstract concepts, and the benefits of new technology for implicit body manipulation

**DOI:** 10.3389/fpsyg.2014.00757

**Published:** 2014-08-19

**Authors:** Katinka Dijkstra, Anita Eerland, Josjan Zijlmans, Lysanne S. Post

**Affiliations:** ^1^Department of Psychology, Erasmus University RotterdamRotterdam, Netherlands; ^2^Department of Psychology, Open UniversityHeerlen, Netherlands; ^3^Department of Child and Adolescent Psychiatry, VU University Medical CenterAmsterdam, Netherlands

**Keywords:** embodied cognition, body manipulation, conceptual metaphors, abstract concepts, wii balance board

## Abstract

Current approaches on cognition hold that concrete concepts are grounded in concrete experiences. There is no consensus, however, as to whether this is equally true for abstract concepts. In this review we discuss how the body might be involved in understanding abstract concepts through metaphor activation. Substantial research has been conducted on the activation of common orientational metaphors with bodily manipulations, such as “power is up” and “more is up” representations. We will focus on the political metaphor that has a more complex association between the concept and the concrete domain. However, the outcomes of studies on this political metaphor have not always been consistent, possibly because the experimental manipulation was not implicit enough. The inclusion of new technological devices in this area of research, such as the Wii Balance Board, seems promising in order to assess the groundedness of abstract conceptual spatial metaphors in an implicit manner. This may aid further research to effectively demonstrate the interrelatedness between the body and more abstract representations.

## Introduction

Imagine you are reading a story in which someone turns up the volume on his car radio while in real life you are closing the top of a soda bottle. Would these two things (reading about an action and performing a very similar action) influence each other? Research suggests they would. When you are closing the bottle, you are likely to read the words “turn up” faster than if you were opening the bottle (Zwaan and Taylor, 2006). It appears that a rotation of your hand that is congruent with an implied rotation in a sentence facilitates the speed with which relevant parts of the sentence are being processed.

This happens because readers make an elaborate mental representation of what they read that is similar to their experience in real life. How close is this connection between actions on one hand and cognitive processes, such as reading, on the other? Does it only apply to identical actual and implied movements, or does the connection extend beyond these mappings, for example to **abstract concepts** that do not imply movement at all? According to the **embodied cognition** approaches such connections also exist.

KEY CONCEPT 1. Abstract concepts Abstract concepts refer to entities that have no physical or spatial constraints because they have no direct representation in the physical world. It does not exist at a particular time or place but as a type of thing. Examples of abstract concepts are emotions, metaphors, and abstract actions (e.g., thinking).

KEY CONCEPT 2. Embodied cognition Cognition is shaped by aspects of the body, like the motor system, the perceptual system and interactions with the environment. Cognitive concepts have specific neural underpinnings meaning that the reconstruction of an earlier experience involves activation of the same brain systems as those during the original experience. This reactivation process is called “sensori-motor simulation.”

Theories on embodied cognition are gaining importance in the field of psychology and beyond (Pecher et al., 2011; Wilson-Mendenhall et al., 2011; Dijkstra and Zwaan, 2014; Glenberg et al., 2014). In contrast to earlier theories on cognition that consider processing and storage of incoming information to take place in an abstract, symbolic manner, embodied cognition theories focus on the body as being central to shaping the mind (Wilson, 2002). Specifically, cognitive processes are presumed to depend on the sensory-motor system in the brain that reactivates earlier experiences, a process called sensory-motor simulation (Barsalou, 1999). When such an experience is retrieved, neural states are re-enacted from the systems that were relevant for the original experience, such as action and perception systems. Cognition is therefore grounded through simulation (Barsalou et al., 2003; Dijkstra and Zwaan, 2014).

Based on the available empirical evidence that has accumulated over the past decade or so, this link between actions and representations of **concrete concepts** has been well established. Recently, more critical points of view have been articulated regarding the specificity of the embodied cognition approach and the boundaries of phenomena that can be explained with this approach. One argument is that embodied cognition research has merely demonstrated that “thoughts and actions go together” but not that the body is essential in carrying out cognitive tasks (Mahon and Caramazza, 2008; Wilson and Golonka, 2013). Another argument is that any effects of grounding are taken as positive evidence for embodiment even if they are different or oppose one another (Willems and Francken, 2012). Rather than making general predictions regarding the involvement of sensory-motor systems in cognitive processes that back all findings, the hypotheses should be more specific and the explanation should focus more on underlying mechanisms of embodiment. A third argument concerns support for the claim that similar connections exist between actions and representations of abstract and concrete concepts (Mahon and Caramazza, 2008; Pecher et al., 2011; Maglio and Trope, 2012). This claim has been challenged because abstract concepts, in contrast to concrete concepts, refer to entities that have no physical or spatial constraints, hence a direct mapping of an abstract concept, such as “democracy,” with a sensory-motor domain is problematic. If abstract concepts without a direct representation in the physical world cannot be physically interacted with, how can they ever be represented through simulation (Mahon and Caramazza, 2008)?

KEY CONCEPT 3. Concrete concepts Concrete concepts refer to something that is present in the physical world, such as a tree in a forest. This means that these concepts have physical or spatial constraints. A tree can grow in a forest but not on the moon. Concrete concepts include but are not limited to physical objects in the world. Concrete actions, such as kicking or smiling, are also examples of concrete concepts.

Addressing these arguments with research involving specific hypotheses regarding the role of the body in the way we think and how abstract concepts are grounded is essential in order to be able to “take the next step” in embodied cognition research. The role and impact of the body on cognitive processing have to be specific enough in order to test falsifiable hypotheses. One way to do this, is to specify in each study *when* and *how* embodiment occurs (Willems and Francken, 2012). Moreover, it is important to determine the role of the body in cognitive processes in as much detail as possible. This can be done by asking questions, such as: Are sensory-motor processes necessary for cognitive processing, sufficient for cognitive processing, neither necessary nor sufficient or are sensory-motor processes only needed for deep conceptual processing (Fischer and Zwaan, 2008)? The answers may differ depending on the task being used, and the frame of mind an individual has on a given point in time (Maglio and Trope, 2012). The groundedness of abstract concepts can be evaluated with empirical evidence from studies that have examined abstract concepts as instantiations of concrete concepts in a situation (Lakoff and Johnson, 1980, 1999).

Abstract concepts, such as “democracy” are considered to have an indirect basis in the sensory-motor system as representations of situations that are created from different individual experiences, such as “voting in a voting booth” (Barsalou and Wiemer-Hastings, 2005). Thus, abstract concepts can be understood in terms of concrete concepts through metaphorical associations with concrete domains of experiences. Research has provided empirical evidence for this mapping between abstract concepts and concrete experiences. **Orientational metaphors** provide a spatial orientation for an abstract concept, which can be vertical (down-up), horizontal (left-right) or sagittal (front-back). For some metaphors, there is a physical basis, for example “more is up” because stacking items vertically coincides with a higher quantity of those items. The metaphor “power is up” has a more indirect physical basis, as a result of experiences of statistical regularities that one encounters from infancy onward where power is exerted by someone with greater height (parent-infant, teacher-child).

KEY CONCEPT 4. Orientational metaphors Orientational metaphors are metaphors in which concepts are spatially related to one another. For example, when we speak of feeling “up” or “down,” or when we think of the future being “in front of” and the past “behind” us. Orientational metaphors are exceptionally useful in research because they are plentiful, used in a variety of ways, and easy to represent and manipulate in experiments.

Other mappings between abstract concepts and concrete experiences have been established by bodily manipulations as well. The **conceptual metaphors** “right is more” and “up is more” were activated by having participants move in a chair along x- and y- axes with higher numbers being generated when moving left-to-right and upwards (Dehaene et al., 1993; Hartmann et al., 2012). Other studies examined how orientational metaphors could be combined with the way emotions are represented as “positive is up” (Crawford et al., 2006; Casasanto and Dijkstra, 2010). In those cases, the mapping between the abstract concept and concrete domain is even more complex, because it is based on an association of emotional life experiences and vertical motion. Participants who moved marbles upward or downward with their hands activated the metaphor of “positive is up” and “negative is down” by retrieving positive memories when moving upward and negative when moving downward (Casasanto and Dijkstra, 2010). The “positive is up,” “negative is down” representation of the conceptual emotional metaphor has its origin in life experiences, where we cheer when we are happy and sit down with our head down in our hands when we are sad. It is therefore remarkable that this metaphor is still activated when an unrelated movement (depositing marbles upward or downward in a container) is being performed.

KEY CONCEPT 5. Conceptual metaphors Abstract concepts are understood in the context of concrete experiences. For example, the metaphor “Life is a journey” connects the abstract concept of life to experiences during which one went on a journey and knowing that it has an element of time and destination to it. Because of such a concrete experience, the sensory-motor system forms the basis of the representation of the abstract concept.

Another abstract concept for which complex mappings with concrete experiences exist is “time.” Time has been represented along the horizontal axis with the past being associated with left and the future with right (Santiago et al., 2007; Ulrich and Maienborn, 2010). Santiago et al. (2007) found that people are faster to categorize words as belonging to the past or the future when words are categorized as the past with a left-hand and future words with a right-hand response. This time metaphor can be modulated by cultural differences in language representations. For example, speakers of Mandarin use vertical terms to talk about time and therefore responded more quickly to vertical representations of time compared to speakers of English who think about time in horizontal spatial terms (Boroditsky, 2001). Abstract concepts may not always be directly grounded through interactions with the world but have their basis in instantiations of concrete experiences and co-occurrences with a certain representation. These mappings between abstract concepts and their more concrete representations are dynamic and can not only be learned over time but also change when co-occurrences change.

This discussion of research that demonstrates how abstract concepts are grounded in action and perception provides insight into the *what* and *when* conditions under which grounding occurs. All studies conducted from an embodied cognition perspective, demonstrated an effect of the body (whole body or hand movement) on task performance with the response following the body manipulation, not the other way around. The question remains, however, if there are boundaries with regard to the groundedness of abstract concepts. It is feasible that the sensory-motor system becomes less involved with a higher abstractness of a concept (Clark, 1999). We will address this issue in more detail by reviewing several studies on a particularly abstract conceptual metaphor that may pose a challenge to demonstrate effects of embodiment with: the spatial political metaphor.

## Spatial orientation, body manipulation, and political metaphors

The representation of politics along a horizontal axis originates from the way the French Legislative Assembly (established in 1791) was spatially organized in the assembly room with conservatives situated on the right and liberals on the left. This spatial organization has resulted in the construction of the abstract political concept of the right equaling the conservative end of the spectrum and the left as the liberal or progressive end. The abstractness of this metaphor may differ depending on the country it is being used in. In the United States, political debates are broadcasted with liberals on the viewer's left and conservatives on the viewer's right. In the Netherlands, on the other hand, the political left/right distinction is represented as a continuum of several parties, suggesting a more subtle spatial array of left to right. Therefore, even as the actual left-right seating arrangements have largely been abandoned the metaphor for political left and right remains in most western countries (Goodsell, 1988). Can this abstract political concept with such an obscure experiential basis still be activated with a manipulation of the body?

Several studies have examined the possibility of this activation in several countries that vary in the way in which political parties are represented (Oppenheimer and Trail, 2010; Van Elk et al., 2010; Dijkstra et al., 2012; Farias et al., 2013). Oppenheimer and Trail (2010) demonstrated the activation of the political metaphor in three experiments with different body manipulations (squeezing a hand-grip with the right or left hand, sitting on a chair tilted to the left or right, and clicking on visual targets on the left or right side of a screen). A manipulation to the left resulted in a higher agreement with Democrats on political issues but a manipulation to the right did not result in higher agreement with Republicans. Van Elk et al. (2010) found support for the groundedness of **political metaphors** in the Netherlands, where not two, but ten political parties are represented in the political landscape, indicating a true continuum of left to central, and from central to rightwing parties. Participants were manipulated to use their left or right hand to respond to acronyms of political parties and Dutch broadcasting companies, or to respond with the same hand and to stimuli presented on the left or right side of the monitor. Overall, the authors found that participants were faster to respond with their hand that was congruent to the political affiliation of a shown acronym of a political party than with their incongruent hand. However, the effects varied across experiments, sometimes demonstrating congruency effects only for rightwing and sometimes only for leftwing parties. In other words, the association between spatial orientation and politics affected online judgments of political acronyms but the effects were not consistent across experiments. In a third study, researchers demonstrated that the political conceptualization of left to right is also apparent when tested with an auditory measure (Farias et al., 2013). Participants judged conservative words to be louder to the right ear than to the left ear and socialist words to be louder to the left ear than to the right ear.

KEY CONCEPT 6. Political metaphors “Politics” is an abstract concept that can be understood in the context of more concrete experiences. For example, the terms “left” and “right” are used in politics as equivalent of liberalism (or progressiveness) and conservatism respectively. This representation of politics on a horizontal axis originates from the way the French Legislative Assembly was spatially organized in the assembly room.

These studies all demonstrated an association between the abstract concept of the political right and left and the concrete concept of spatial right and left. Although the studies support the idea that the relationship between abstract concepts and concrete domains is integrated in multiple modalities, a problematic element in several of the experiments was that the congruency effects were not consistent for both leftwing and rightwing affiliations. Could this be indicative of a boundary limitation of the groundedness of abstract concepts? Not necessarily. Effects were demonstrated, even if they were asymmetrical. Also, an alternative explanation of these findings could be that participants were aware of the manipulation, which could have affected their response. The use of the hands, ears, and visual fields could have been obvious to them as left/right manipulations and thus have given away what the experimenters were after. Another possibility is that political metaphors are more complex in countries without a political dichotomy, such as the Netherlands, because the political landscape consists of multiple parties that form a left-to-right continuum, rather than a left and a right pole. The political metaphor may still be activated then but constitute a more complex mapping with parties in the continuum and therefore yield more inconsistent results.

Given these problematic aspects of the studies discussed above, a better, more subtle way to manipulate the body, is needed. A more implicit manipulation of the body may yield more consistent results and hide the true purpose of the task at the same time. A particularly effective way to do that is to make use of devices that enable such implicit manipulations. A promising line of investigation in this respect is research that uses new technology to measure changes in the body in a surreptitious manner. This technology can overcome some of the problems associated with the earlier studies on political metaphors and provide an effective tool to both manipulate and assess the activation of conceptual metaphors.

## The wii balance board as a tool to implicitly manipulate the body

New technologies used for leisure time activities at home, such as the LEAP-motion (i.e., an infrared device that detects and reacts upon hand movements; Weichert et al., 2013), the Wiimote (i.e., a wireless input device that uses Bluetooth technology), and the Wii Balance Board (WBB), have become increasingly popular among researchers to empirically assess behavior in the lab. Their popularity is based on the facts that most people are already familiar with them, they are relatively inexpensive, and provide precise and useful data as outcome measures.

**Figure 1:**
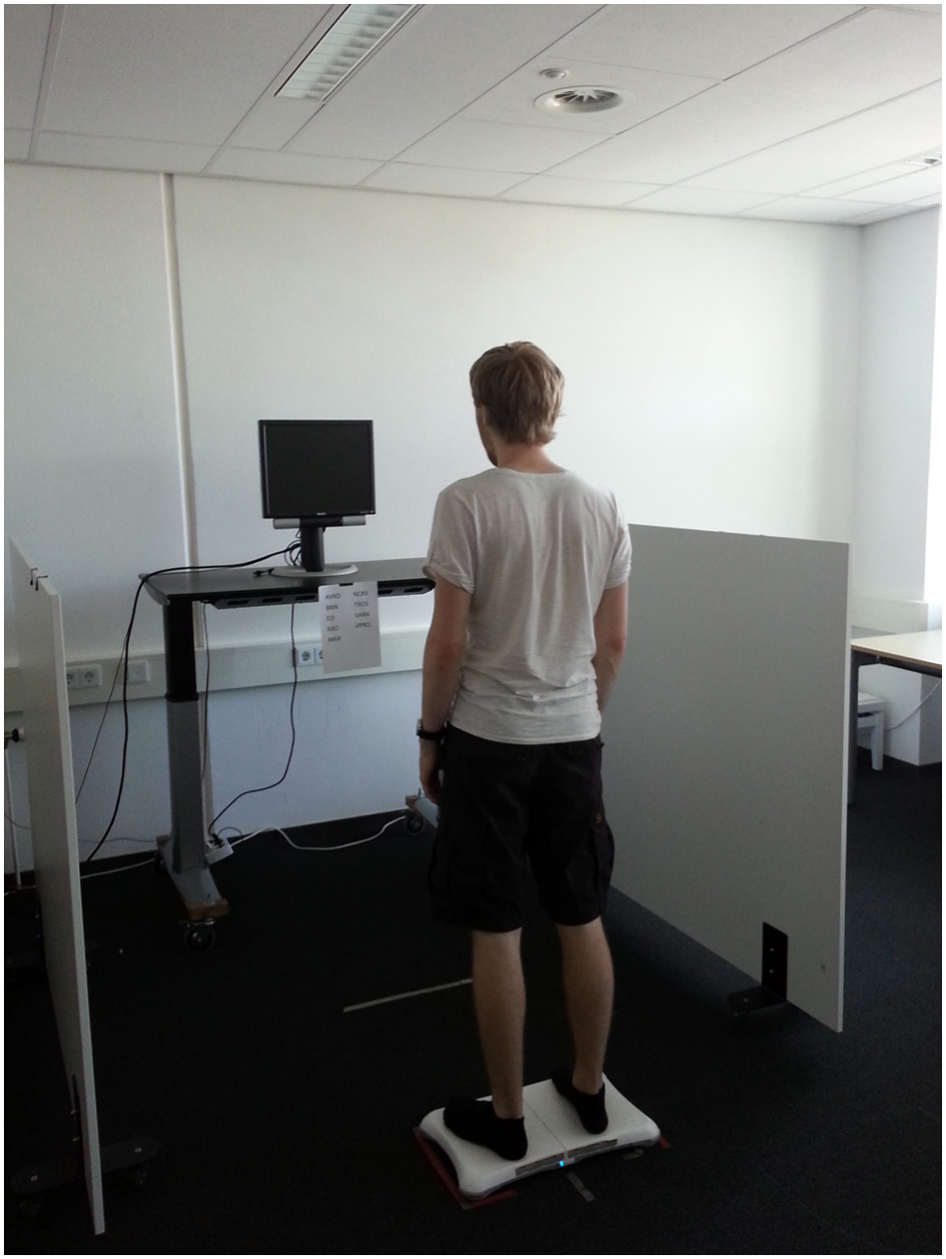
**Wii Balance Board set-up**.

Of these new technologies, the WBB has been used the most in research so far (see Figure 1). The board is 20.1 inches wide, 12.4 inches long, and weighs approximately eight pounds. The four transducers, one in each corner of the board, are used to assess weight distribution and to detect even very small changes in the distribution of participants' center of pressure (COP). Data are sampled at a rate of 33 Hz and the WBB connects to a PC via Bluetooth. The measurements of participants' COP produced by the WBB are as reliable and valid as those produced by other platforms commonly used to assess posture (Clark et al., 2010). In contrast to these platforms, the WBB is far less expensive, and portable, and therefore an attractive alternative for researchers interested in measuring posture. The WBB data contain the COPs of every sample point in the form of X (right-left) and Y (front-back) coordinates. Positive X and Y coordinates indicate that the COP is more to the right and front than the middle of the WBB, while negative X- and Y-values indicate that the COP is more to the left and back. Depending on what a researcher wants to measure, these coordinates can be used to calculate a dependent measure in a program such as Excel or SPSS. For example, if one wants to know whether participants lean to a side, one can look at the corresponding X or Y coordinates and compare COPs across experimental conditions. Another possibility is to see whether people move more in a certain condition (stability of or sway in posture) by measuring the shifts in direction of change in COPs along one of the axes (X-axis for left-right, Y-axis for front-back).

There are several ways in which the WBB has been utilized in behavioral research. These studies vary in the population being tested, from healthy younger adults (see discussion below) to healthy older adults (Koslucher et al., 2012), individuals with Autism Spectrum Disorder (Travers et al., 2013), and stroke victims (Nijboer et al., 2014). They also vary in what is being measured, such as postural stability, sway, or the influence of posture on cognitive processes, such as the activation of conceptual metaphors (Eerland et al., 2012).

Posture has been investigated as a WBB outcome measure in several studies (Eerland et al., 2011; Zwaan et al., 2012; Schneider et al., 2013). In the study by Eerland et al. (2011), participants moved sideways in reaction to an arrow that appeared on the screen. Additionally, the researchers examined whether people leaned more forward in reaction to pleasant pictures (approach behavior) and more backward in reaction to unpleasant pictures (avoidance behavior). This turned out to be the case. In another study (Zwaan et al., 2012), participants moved sideways to indicate whether the sentence they just read was sensible or not. Some action sentences implied a forward-leaning body posture (e.g., The man petted the little dog), while other sentences implied a backward-leaning body posture (e.g., The boy looked up at the clock tower). The hypothesis that posture would be influenced by the described actions as assessed with the WBB was supported. More recently, the WBB was used in a study to assess the influence of ambivalence on body movements (Schneider et al., 2013) based on the idea that when people have ambivalent feelings, they hold positive as well as negative evaluations of an object or issue. Indeed, participants were found to engage in side-to-side movements when experiencing ambivalence.

These results suggest that the WBB is a useful instrument as an outcome measure that also provides very precise data for analysis. Basically, any deviation from the COP is recorded and can be included in the data set, no matter how subtle this pressure shift is. Moreover, the WBB can be used to manipulate posture. This means that it is possible to trick people into believing that they hold a certain position while they are actually not. This is accomplished by having participants think that they are standing upright while in fact they are standing slightly tilted to the right or the left. This manipulation is so subtle that participants are not aware at all that they are standing sideways instead of upright.

The first study to demonstrate such an implicit activation of abstract concepts, built on the mental number line theory (Restle, 1970) as an example that people mentally represent numbers along a line, with small numbers on the left and large numbers on the right. The idea behind the study was that having people lean slightly to the left or the right would activate this mental number line. Indeed, support was found for the idea that surreptitiously leaning to the left activated relatively smaller numbers than surreptitiously leaning to the right (Eerland et al., 2011).

**Figure 2:**
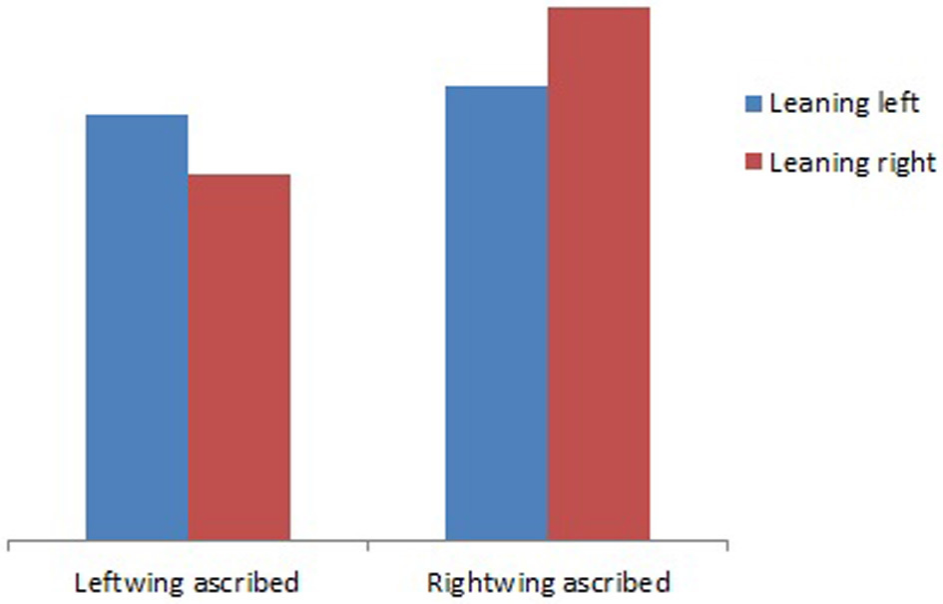
**Ascribed statements to left- and right-wing parties by leaning position**.

The second study examined the effect of an implicit body posture manipulation with the WBB, in a similar way as in the previous study but then with the political affiliation metaphor along a horizontal axis (Dijkstra et al., 2012). Specifically, the subtle body manipulation on the WBB was examined to assess its effects on political party evaluations. This was done in a Dutch political party environment of 10 parties that can be placed on a left-right continuum. Just as in the Eerland et al. (2011) study, participants thought they stood upright on a WBB while in fact they stood slightly tilted to the left or the right. They were then asked to ascribe general political statements (that could not directly be attributed to one of the political parties in the Dutch House of Representatives) to one political party. The manipulated body position was expected to affect one's political attribution such that standing somewhat to the right would result in attribution of the statement to a political party on the right and an attribution to a party on the left when leaning to the left. The results (see Figure 2) indicated that there was an interaction of leaning direction with party attribution as expected, such that there was a congruence effect of leaning direction with left-wing party attribution.

This study is a good demonstration of how an abstract metaphor that has a complex mapping with concrete associations can be activated effectively with a sensori-motor manipulation of the body. The use of the Wii Balance Board facilitated the use of the whole body, rather than parts of the body, as was the case in earlier studies on political metaphors. Moreover, the manipulation was implicit because participants were under the impression that they were standing in the middle of the balance board even though they were not. They were not aware of the fact that their body posture could and would affect their evaluations and most likely did not consciously perceive proprioceptive feedback that they were leaning to the left or the right. Instead, they relied on the visual feedback from the screen. None of the participants noticed the manipulation. Since they were under the impression that they had to maintain their balance in the middle of the board. Such a manipulation makes the WBB an appropriate device for research on the implicit activation of concepts.

Another important component of the study was that the political statements were general and could be attributed to either conservative, liberal, or progressive parties. The attribution was affected therefore by the manipulation of the body, not by party-based content of the statements. The task was also very different from the ones in the previous studies on the political metaphor. In the Oppenheimer and Trail study (2010), the tasks involved a rating of the level of agreement with the leftwing or rightwing political party whereas the tasks in the experiments of Van Elk et al. (2010) involved response times for which the answers were either correct or incorrect. In contrast, the studies conducted by Farias et al. (2013) and Dijkstra et al. (2012) had more subtle task demands for which the answer clearly depended on the manipulation of the body and no answer could be correct. Moreover, pretests were conducted to create a reliable set of stimuli, socialist-referent words and conservative-referent words in the Farias et al. (2013) study, and a set of statements that were equally likely to be attributed to left-wing or right-wing parties in the Dijkstra et al. (2012) study. Filler questions regarding television programs were added in the latter study to steer the focus away from an exclusive political theme.

We can conclude that the Nintendo Wii-Balance Board (WBB) seems to be a promising tool to investigate left-to-right oriented linguistic metaphors, such as the political metaphor. A main advantage of the WBB is that people can lean one way or the other without even noticing it. This provides the opportunity to investigate other left-to-right metaphors such as emotional valence, the mental number line, or time, while participants are not aware of the fact that their posture is being manipulated. It is entirely possible that given neutral prompts, people might report more positive memories or judge pictures to be more positive when leaning to the right than when leaning to the left. It is conceivable, that these effects are bidirectional. Previous research has demonstrated changes in body posture when participants were primed with concepts, such as “pride” and “shame” (Oosterwijk et al., 2009). Similarly, for the political metaphor, bidirectionality could be demonstrated if participants would lean to the right when being primed with statements reflecting right-wing political issues and to the left when prompted with statements reflecting left-wing issues.

The two studies on conceptual metaphors manipulating posture with the WBB not only provide evidence that this device can be used effectively to manipulate people's judgments, it also supports the idea that even abstract conceptual metaphors are activated when manipulating body position. Apparently, we understand abstract concepts both through concrete experiences and learned associations even if the experiential basis is limited and the mapping is complex. Subtle manipulations of the body without participants being aware of it may work for other conceptual metaphors as well that have no or a limited experiential basis.

Given the promising possibilities of the WBB to manipulate posture, further empirical research could address the issue of learning. If leaning to a side can influence judgments about left/right-statements because the metaphor is grounded (Dijkstra et al., 2012), then it may also work in another direction. Perhaps learning ambiguous material can be directed a certain way by manipulating posture in a congruent direction. Leaning to the left could, for example, facilitate learning which political parties are left-wing parties. Research is needed to investigate this influence of posture on learning, because it might very well be that the subtle manipulation is too implicit to promote explicit learning. It is also interesting that the effect of posture manipulation was only found for the actual left and right positions of parties, and not for what people thought were left and right parties (Dijkstra et al., 2012). The most logical explanation for this finding is that people had difficulties in reporting the position of the party on a complex grid (see Dijkstra et al., 2012). However, it could also mean that this is an issue that still has to be investigated. If it is important for the influence of posture what people do or do not explicitly know about a subject, it will probably have implications for studies using this manipulation to examine learning outcomes.

## Conclusions

One of the main contributions of research on the groundedness of abstract concepts using new technologies such as the WBB, is that body manipulations can be implemented in a subtle manner that do not alert participants as to what is happening or is supposed to happen. The WBB provides very precise measurements of participants' center of pressure. This can be kept constant during a manipulation by having participants look at their body as a mark on the screen which helps them to keep themselves in the required location. This affords very specific and credible feedback to the participant that their body position is where it should be and how to remain in this position even though in fact their body is tilted to the right or the left. Inclusion of these technologies in future research may be valuable because the activation of other complex metaphors could be assessed this way, both as a tool to collect posture data (along the x- and y-axes), and as a tool to manipulate posture in different ways (along the sagittal axis or by creating imbalance).

We do not claim that these abstract concepts are grounded in the sense that motor activation is necessary for understanding the concept, but research with the WBB does show that it is more than just co-activation. When encountering an ambiguous situation, one uses all available resources to resolve the ambiguity. For instance, when reading an ambiguous political sentence (i.e., it is not clear to which party the statement belongs), people might use the unconscious proprioceptive feedback of their body when evaluating the statement and attributing it to a political party. The body clearly plays a role here, moving beyond the enrichment of those concepts by facilitating a choice within the relational context in which conceptual processing takes place.

How far do these effects go? According to Maglio and Trope (2012), there are boundaries to such effects of embodiment. They stipulate that these effects only occur when a certain frame of mind is created in the participant. Participants who were manipulated to think at a higher, abstract level were less responsive to contextual bodily cues than when they were encouraged to think in a more concrete manner. The abstract thinking manipulation thus prevented the contextual proprioceptive feedback from affecting judgments. The issue we encountered in the discussion of research on political metaphors, however, was that the effects are not always consistent. The participant's frame of mind (abstract vs. concrete) did not seem to be the issue here. It was rather the effectiveness of the manipulation and possible awareness among participants that may have influenced some of the outcomes.

In our view, future research should therefore focus more on examining in detail *how* and *when* this activation takes place (Willems and Francken, 2012), and for which tasks specifically. Possibly, effects are stronger when the whole body shifts to the left or the right instead of parts of the body. Future research should also investigate if these patterns replicate for similar manipulations but different concepts, or similar concepts but different tasks. The next step would be to do this for other metaphors with complex mappings. The outcomes of these studies should not be merely an addition to the current pile of evidence, but can instead bring us closer to a deeper insight into the mechanisms underlying the grounding of abstract concepts. Sensory-motor activation is as applicable to abstract concepts as to concrete concepts, particularly for tasks that involve a certain level of ambiguity. Sensory-motor processes do not seem to “tweak” the results of cognitive processing but are part of the decision process that leads to a response. So far, sensory-motor grounding has been reliably demonstrated for abstract concepts. Further research could reach the limitations of embodiment or support the view that sensory-motor representations are necessary and/or sufficient for cognitive processing. Either way, it will narrow down what the role of the body in conceptual processing is.

### Conflict of interest statement

The authors declare that the research was conducted in the absence of any commercial or financial relationships that could be construed as a potential conflict of interest.
